# Deciphering the genetic structure of the Quebec founder population using genealogies

**DOI:** 10.1038/s41431-023-01356-2

**Published:** 2023-04-04

**Authors:** Laurence Gagnon, Claudia Moreau, Catherine Laprise, Hélène Vézina, Simon L. Girard

**Affiliations:** 1 Département des Sciences Fondamentales, Université du Québec à Chicoutimi, Saguenay, Québec G7H 2B1 Canada; 2 Centre Intersectoriel en Santé Durable (CISD), Université du Québec à Chicoutimi, Saguenay, Québec G7H 2B1 Canada; 3 Centre Intégré Universitaire en Santé et Services Sociaux du Saguenay–Lac-Saint-Jean, Saguenay, Québec G7H 7K9 Canada; 4 Département des Sciences Humaines et Sociales, Université du Québec à Chicoutimi, Saguenay, Québec G7H 2B1 Canada; 5 Projet BALSAC, Université du Québec à Chicoutimi, Saguenay, Québec G7H 2B1 Canada; 6 Centre de Recherche CERVO, Université Laval, Québec, Québec G1V 0A6 Canada

**Keywords:** Population genetics, Consanguinity

## Abstract

Using genealogy to study the demographic history of a population makes it possible to overcome the models and assumptions often used in population genetics. The Quebec founder population is one of the few populations in the world having access to the complete genealogy of the last 400 years. The goal of this study is to follow the evolution of the Quebec population structure over time from the beginning of European colonization until the present day. To do so, we calculated the kinship coefficients of all ancestors’ pairs in the ascending genealogy of 665 subjects from eight regional and ethnocultural groups per 25-year period. We show that the Quebec population structure appeared progressively in the St. Lawrence valley as early as 1750 with the distinction of the Saguenay and Gaspesian groups. At that time, the ancestors of two groups, the Sagueneans and the Acadians from the Gaspé Peninsula, experienced a marked increase in kinship and inbreeding levels which have shaped the structure and led to the contemporary population structure. Interestingly, this structure arose before the colonization of the Saguenay region and at the very beginning of the Gaspé Peninsula settlement. The resulting regional founder effects in these groups led to differences in the present-day identity-by-descent sharing, the Gaspé and North Shore groups sharing more large segments and the Sagueneans more short segments. This is also reflected by the distribution of the number of most recent common ancestors at different generations and their genetic contribution to the studied subjects.

## Introduction

Founder populations have been particularly helpful in demonstrating how past demographic events have shaped present-day genetic structure and its consequences on human health [1–3]. Studying the past demographic history of a population often relies on current genetic data and models of ascending genealogical trees [4, 5]. However, developing efficient methods for inferring the underlying genealogy has proved challenging [6, 7] or requires lots of contemporary and ancient genomes data [8]. To avoid using such assumptions, one would need the complete genealogy of the population. Few populations in the world have access to such genealogical data [9–11]. The Quebec province of Canada relies on the BALSAC population register, a large collection of linked data from parish records, to reconstruct the genealogy of the vast majority of Quebecers, mostly of French Canadian descent, but also of other origins, since the foundation of the colony in the 17th century until recent times [12]. This invaluable resource allows the detailed mapping of the population structure over time. Indeed, it has been shown that the genealogical lines covering the last 400 years explain most of the present-day genetic structure of the Quebec population [13, 14].

This study will focus on Quebecers genealogically anchored into five regions (from west to east): the Montreal and Quebec City areas, the Saguenay-Lac-St-Jean (Saguenay) and North Shore regions, and the Gaspé Peninsula (Gaspé) where four subgroups were sampled (Acadians, French Canadians, Loyalists, and Channel Islanders). Most Quebecers of French Canadian ancestry are descendants of around 8500 settlers who came predominantly from France between 1608 and 1760 [15]. These European newcomers first settled in Quebec City (1608) and Montreal (1642) which are now two major urban regions (Supplementary Fig. S1A) and along the shores of the St.Lawrence river. Following the British Conquest of 1760, French immigration decreased dramatically, and the French-speaking population expanded mostly through natural increase. Population growth led to the colonization of new regions, including more remote and isolated regions, favoring population subdivision [13].

Permanent European settlement in Gaspé began during the second half of the 18th century with the arrival of Acadians, who escaped deportation by the British [16]. They were soon joined by English-speaking United Empire Loyalists who chose to remain under British rule after the American Declaration of Independence in 1776. From 1830–1840, many Quebecers of French Canadian ancestry from the lower part of the St. Lawrence valley also settled in the Gaspé peninsula [16]. At the same time, a fourth group, inhabitants of the Channel Islands, came to Gaspé for the fishing industry.

The settlement of Saguenay started in 1838 with founders mostly coming from the neighboring region of Charlevoix which was colonized earlier by the end of the 17th century. The Saguenay population size underwent a 25-fold increase between 1861 and 1961, mostly due to a high birth rate [17, 18], while the whole Quebec population increased only 5-fold. The western part of the North Shore was colonized by ancestors who came from the Charlevoix and Bas-St-Laurent regions [19] while the eastern part pioneers were mostly fishermen from Iles-de-la-Madeleine and Gaspé.

Genetic data is often used to study the contemporary population structure [20] and we have previously shown that the genetic structure of the Quebec population is well correlated with the one inferred using genealogical measures [13, 21, 22]. However, genealogies are an invaluable tool to study how the population structure was shaped in the past generations. The goal of this study was to follow the evolution of the Quebec regional population structure from its colonization until the present day. To do so, we looked at the kinship and inbreeding levels of all ancestors in the genealogies. We also deciphered the population fine structure inferred with present-day genetic identity-by-descent (IBD) sharing using genealogical measures.

## Subjects and methods

This study was approved by the University of Quebec in Chicoutimi (UQAC) ethics board. Written informed consent was obtained from all adult participants.

### Cohort

The data consist of 579 subjects from the Quebec Regional Reference Sample and 86 unaffected subjects from the Saguenay-Lac-St-Jean asthma familial cohort (Supplementary Fig. S1A and Table 1) [13, 21, 23]. The subjects are distributed in five regions and eight groups (based on geographical and ethnocultural criteria) of the province of Quebec: the Montreal and Quebec City areas, the Saguenay and North Shore regions, and the Gaspé Peninsula. For the latter, four subgroups were sampled, namely Acadians, French Canadians, Loyalists, and Channel Islanders. Subjects were sampled regardless of their proportion in the population. To ensure regional connection, subjects needed to have their four grandparents born in the Quebec province and one or two parents born in the particular region except for the Montreal area where only first criterion (the four grandparents) was applied. The four ethnocultural subgroups of Gaspé were self-reported. A strong correspondence between ancestral origins traced in genealogies and self-reported origins was found in a previous study [24]. Genotyping and ascending genealogical data are available for the 665 subjects.

**Table 1:** Regional and ethnocultural contemporary groups’ description.

Group	Abbreviation	Sample Size	Average year of Parents’ Marriage
Montreal Area	MTL	138	1950
Quebec City Area	QUE	70	1945
Saguenay-Lac-Saint-Jean	SAG	86	1977
North Shore	NSH	47	1949
Gaspé French Canadians	GFC	97	1945
Gaspé Loyalists	GLO	71	1939
Gaspé Channel Islanders	GCI	67	1941
Gaspé Acadians	GAC	89	1942

### Genotyping data and genomic analyses

Genotyping of the 665 subjects was conducted on Illumina Omni Express (~740,000 SNPs) and Illumina Omni 2.5 chips (~2.5 M SNPs) chips. Both chips have been merged to keep only common SNPs (702,216). Quality control filters were applied at the individual and SNP levels using PLINK software v1.9 [25]. We retained subjects with at least 98% genotypes among all SNPs. At the SNP level, we retained SNPs with at least 98% genotypes among all subjects, located on the autosomes and in Hardy–Weinberg equilibrium *p* > 0.001 (calculated on the whole cohort), yielding 659,219 SNPs. Closely related subjects (first cousins, kinship coefficient ≥ 0.0625) were eliminated to avoid bias in the population structure analysis, yielding a final sample size of 665 subjects (Table 1). A principal component analysis (PCA) was performed on SNPs with a minor allele frequency of at least 5% and after pruning to remove SNPs in LD (96,915 SNPs left) using PLINK software to confirm that our final dataset reflects the previously described Quebec population structure [13, 21] (Supplementary Fig. S2).

The assessment of pairwise IBD segments was performed using refinedIBD software v17Jan20 [26] on phased genotypes (done using Beagle software version 18May20.d20). This software was selected for its power and accuracy in detecting IBD segments [20, 26]. Only segments of 2 cM or more and with a LOD score greater than 3 were retained.

### Genealogical data and analyses

Genealogical data were obtained through the BALSAC project [12]. Ascending genealogies were reconstructed for the 665 (contemporary) subjects for whom we have genotype data with average completeness of at least 60% up to the tenth generation for all groups, except for the Loyalists and Channel Islanders of Gaspé (explained in part by their later time of arrival in Quebec), consistent with previous results [13] (Supplementary Fig. S3). The completeness is the proportion of ancestors found at each generation in the genealogy compared to the maximum possible number of ancestors. Information on the parents’ year (±5 years for confidentiality concerns) and region of marriage or if outside Quebec, country of origin was obtained for 94,076 distinct individuals (ancestors and subjects) throughout the genealogy. There are 20 regions of marriage in our data (Supplementary Fig. S1B). We inferred the unknown parents’ marriage years as being the children or grandchildren parents’ marriage year minus 30 or 60 years, the average time between parents’ marriage and their children’s marriages in our data being 32 years. They were grouped into 25-year periods to minimize the parent–child overlap within the same period (2.5% overlap in the 1676–1925 period),

The kinship coefficient at the maximum generational depth was computed using the R GENLIB library v1.1.6 [22] for each pair of ancestors within 25-year periods. Multidimensional scaling (MDS) was performed on the pairwise kinship distance matrix (1-kinship coefficient). Ancestors who had no kinship ties with anybody were removed from this analysis (either founders who have no known parents in the genealogy or ancestors close to founders such as their children or grandchildren who could not be linked to anybody else in the genealogy). For this analysis, the parents’ marriage region was assigned directly to each ancestor and colored according to the 20 regions in Fig. S1B.

The average pairwise kinship and inbreeding coefficients for the ancestors of each group were calculated at the maximal depth for each period. In this analysis we did not assign the region according to the parents’ marriage place of each ancestor, rather, we assigned the ethnocultural or regional group to all ancestors of the contemporary subjects of each group. Consequently, ancestors could be assigned to many groups if they happened to be present in the genealogies of subjects from different groups. In the genealogies, kinship is measured on each pair of individuals (ancestors whose parents were married in each period in our case) and inbreeding is measured within one ancestor and is equal to the kinship coefficient of their parents. Consequently, within the same period of time, the mean kinship for all pairs of ancestors will not necessarily reflect the mean inbreeding for these ancestors since two persons have to mate to produce a child with a certain level of inbreeding. Of course, mating will not happen between all pairs of ancestors in the population. The inbreeding reflects the mating pattern and not necessarily the mean kinship of the population, especially if mating is not random.

Most recent common ancestors (MRCAs) were counted using GENLIB for the subjects’ pairs within groups for each distance in meioses (for example, there are four meioses between two cousins). The minimal distance (the shortest genealogical path) relating both subjects through the MRCA was calculated using GENLIB. A pair can have more than one MRCA as long as no ancestor in the set of MRCAs shares a descendant who is also an ancestor of the pair of subjects. The expected genetic contribution (GC), consisting in summing the transmission probabilities over all genealogical paths connecting an ancestor to a descendant given that parents transmit half of their genome to each child, was also calculated for each MRCA to both descendants using GENLIB. The GC product to both subjects was summed over all MRCAs. Groups were resampled down to 47 subjects 1000 times to avoid size bias. For each bootstrap, we randomly selected 47 subjects in each group and reconstructed the genealogy for this new subjects’ subset. This results in less ancestors in the genealogy and is essential to consider when counting the absolute number of ancestors which depends on the number of subjects.

## Results

### How population structure was shaped

**Fig. 1:**
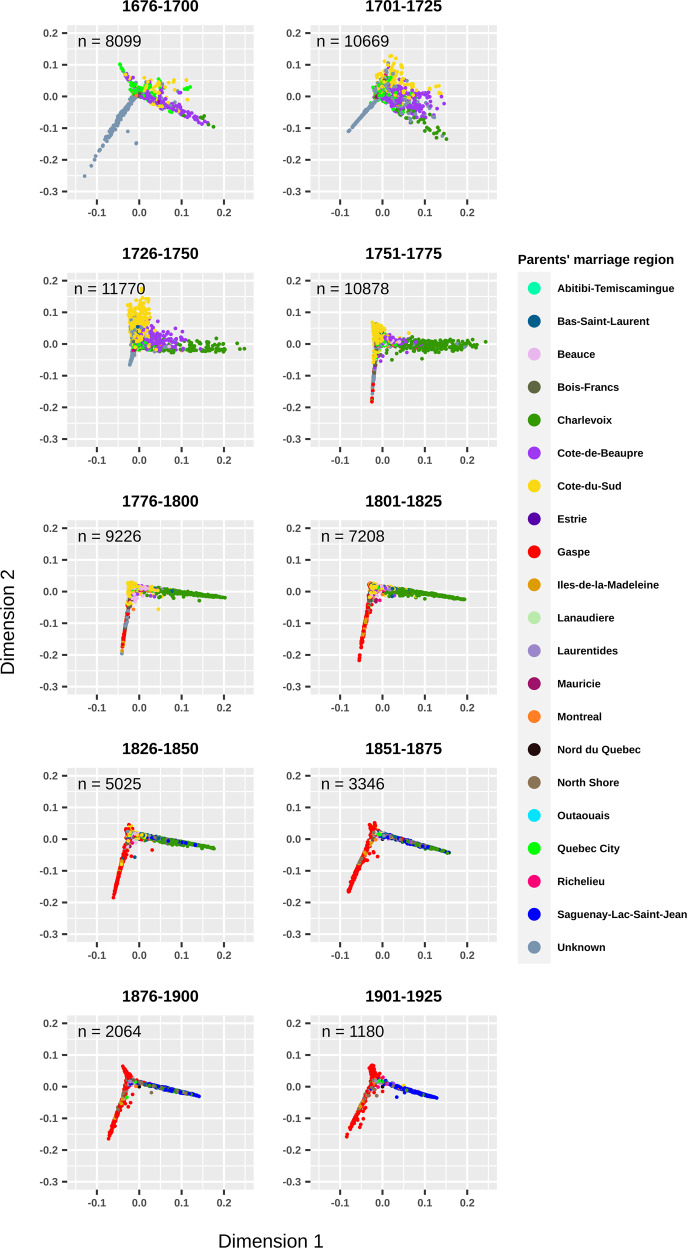
Multidimensional scaling (MDS) of the pairwise kinship coefficients of ancestors of all groups per 25-year period. MDS was performed on the pairwise kinship distance matrix, (i.e., 1-kinship coefficient) of ancestors whose parents were married at each period. The pairwise kinship coefficient was computed using the R GENLIB library at the maximal depth. The external interactive version of this figure is available at https://laugag17.github.io/quebec_founder_pop_interactive_figure/figure2_interactive_graph.html.

The evolution of the Quebec population structure was assessed using pairwise kinship coefficients of all ancestors at the maximum depth for each period (Fig. 1) in the ascending genealogies of the 665 subjects all together. In this figure, we used the parents’ marriage region (Supplementary Fig. S1B) to color each dot (representing an ancestor whose parents married in that period). Before 1750, the ancestors from three regions (Côte-de-Beaupré, Côte-du-Sud, and Charlevoix), progressively differentiated from each other along the *x* axis and from another group of immigrants along the *y* axis, whose parents did not marry in Quebec but whose country of origin is known (see interactive Fig. 1 for countries of origin). These immigrants are mainly coming from Acadia (83%) and a lower proportion from France (5%) and other origins. The present-day population structure (Supplementary Fig. S2) [13] progressively appears as early as 1751–1775 distinguishing Charlevoix and the ancestors of the Gaspé groups (Fig. 1). Note that Gaspé ethnocultural groups can’t be distinguished from the data used in Fig. 1. Prior to 1750, ancestors of each Gaspé group were found almost everywhere on the MDS (Supplementary Fig. S4). Following this period, we can see the effect of the Acadians and Loyalists immigration by the multiplication of ancestors specific to only one Gaspé group and their progressive differentiation along the *y* axis (Supplementary Fig. S4). By 1826–1850, the first ancestors married in Saguenay appeared and the population structure at that time was very similar to the one depicted on the PCA of the present-day subjects (Supplementary Fig. S2) [13].

### Mean kinship and inbreeding over time

**Fig. 2:**
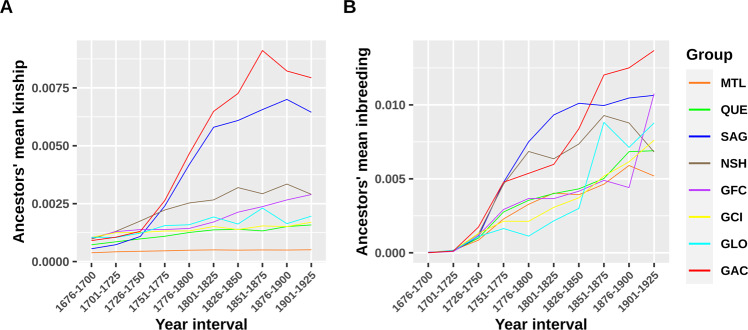
Average kinship and inbreeding coefficients of ancestors of each group per 25-year period. The average pairwise kinship (**A**) and inbreeding (**B**) coefficients for the ancestors of each group were calculated at the maximal depth for each period. The sample sizes are reported in Supplementary Table S1. GAC Gaspé Acadians, GCI Gaspé Channel Islanders, GFC Gaspé French Canadians, GLO Gaspé Loyalists, MTL Montreal, NSH North Shore, QUE Quebec City, SAG Saguenay.

We averaged for ancestors of each contemporary group the genealogical kinship and inbreeding coefficients at the maximum depth for each period (based on the parents’ marriage date) (Fig. 2A and Supplementary Table S1 for counts). From 1750, the GAC and Saguenay ancestors went through a marked increase in averaged kinship compared to the ancestors of the other groups. By 1825, the GAC ancestors’ mean kinship had continued to increase while the Saguenay ancestors had reached a plateau. The ancestors’ mean kinship increase in GAC and Saguenay groups was accompanied by an increase in inbreeding (Fig. 2B). Until 1850, the average inbreeding coefficient at the maximum depth was higher for the Saguenay ancestors. After 1850, the GAC ancestors’ mean inbreeding exceeded the one of the Saguenay ancestors, whereas the latter reached a plateau. The three other Gaspé groups’ average inbreeding coefficient increase was slower at the beginning, but almost reached the Saguenay ancestors’ inbreeding value by 1925. Interestingly, for all groups the increase in inbreeding was more important than the increase in kinship levels.

### IBD sharing and most recent common ancestors

**Fig. 3:**
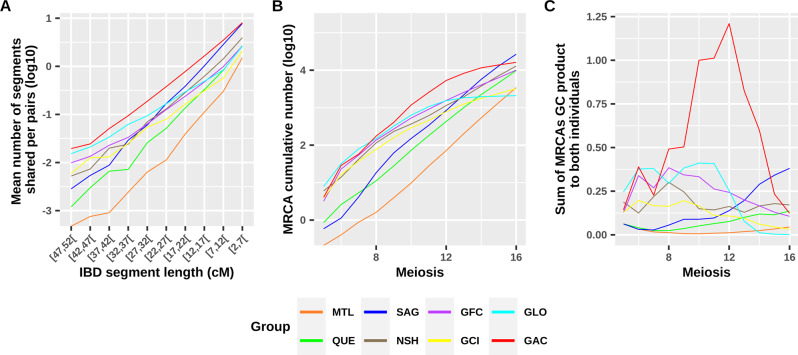
Within groups IBD segment sharing by length as well as MRCA cumulative count and genetic contribution per meiosis. IBD segment lengths (**A**) were binned into 5 cM intervals. The sample sizes for (**A**) are reported in Table 1. The MRCA cumulative count (**B**) and the sum (not cumulative) of their genetic contribution to the contemporary subjects (**C**) are the averages of 1000 bootstraps of 47 subjects. Figure S5 presents the cumulative MRCA counts and genetic contribution until 30 meioses and with the bootstrap intervals. GAC Gaspé Acadians, GCI Gaspé Channel Islanders, GFC Gaspé French Canadians, GLO Gaspé Loyalists, MTL Montreal, NSH North Shore, QUE Quebec City, SAG Saguenay.

For each group, we plotted the mean number of IBD segments shared among subjects’ pairs per segment length (bins of 5 cM) and compared this with the cumulative MRCA counts per meiosis (Fig. 3A, B). Note that MRCAs are not unique so the same MRCAs can appear for many subjects’ pairs and they will be counted each time they appear. These two metrics, one using genetic data and the other using genealogical data, show very similar patterns. Interestingly, the Sagueneans’ pairs shared more IBD segments of short lengths (<22 cM), but less of long lengths (>37 cM) compared to the North Shore and the four Gaspé groups leaving only the urban and older groups (Montreal and Quebec City areas) behind for longer segments. This is also reflected by the cumulative MRCA count until ten meioses (Supplementary Table S2). Inversely, the Gaspé Loyalists (GLO) shared less short segments and more long segments. Note that the MRCA count for the GLO is biased towards the right of the graph (Fig. 3B) since their completeness decreases faster than the other groups (Supplementary Fig. S3). This was also observed for the Gaspé Channel Islanders to a lesser extent.

We also calculated in the genealogies the product of the genetic contributions of each MRCA to both subjects and we summed these products for all MRCAs. Figure 3C presents this GC sum averaged for 1000 bootstraps of 47 subjects (see also Supplementary Fig. S5 for intervals). Note the very high genetic contribution of GAC close MRCAs. For the three other Gaspé groups and the North Shore, closer MRCAs also had a higher GC than those of Montreal, Quebec City, and Saguenay. However, for the latter, the GC sum is higher for more distant MRCAs.

## Discussion

In this study, we show how the Quebec founder’s population structure was shaped over time. We found that the previously described structure differentiating the Gaspé and the Saguenay groups [13] emerged early in the colonization process (1750 or before), almost a hundred years before the colonization of the Saguenay region (1840) [17, 18, 27–29] (Fig. 1). At this time, Saguenay ancestors were mostly located in the Charlevoix region where they had established only two or three generations before. The small number of founding families in Charlevoix followed by a rapid expansion in Saguenay in the 19th century led to changes in the frequencies of alleles and diseases [30]. Similarly, GAC subjects descend from a small number of founding families, but they did not go through a rapid expansion like the Saguenay region. Instead, they mostly married inside their community due to linguistic and cultural barriers present with the other Gaspé groups [31]. Additionally, they were the only ones in the area until 1780 [16]. Both groups’ colonization started with a limited number of founding families implicating that a smaller number of founders explains a higher proportion of the present-day gene pool compared to the other groups [22]. Despite their different subsequent colonization processes, Saguenay and GAC ancestors have a very similar mean kinship increase (Fig. 2A) starting in 1750 when the contemporary population structure appeared. For both Saguenay and GAC ancestors, spouses had higher chances of being related than those of the other groups. However, the average inbreeding was higher among Saguenay ancestors until 1850 when GAC ancestors went through a marked increase which lasted until recent times (Fig. 2B). This is consistent with previous findings showing that close inbreeding of contemporary subjects is the lowest in the province for Sagueneans and the highest for Gaspé [27] (Supplementary Fig. S6). In fact, for both kinship and inbreeding, the Saguenay ancestors reached a plateau at the time the region was colonized (1838) and the expansion started (around 1860) while GAC never went through such a rapid expansion. This increase in inbreeding followed by stabilization among Saguenay ancestors was previously explained by the evolution of nonrandom mating as well as by the evolution of inbreeding resulting from drift [32]. This would need further investigation to understand its implications on the contemporary population. Nevertheless, the GAC and the other Gaspé ethnocultural groups did not reach such a plateau.

The regional fine structure could be observed within groups by comparing IBD sharing patterns (Fig. 3A). To ensure that this fine structure is explained by the recent population history (after the European colonization of Quebec), we focused on large IBD segments which are expected to come from more recent ancestors [33, 34]. The GAC and Saguenay groups share more IBD segments <22 cM than the other groups, in line with previous results on shorter segments [21] and consistent with the higher ancestors’ mean kinship and inbreeding compared to other groups by 1750. However, for long segments >37 cM, the IBD sharing of Sagueneans’ pairs decreases more rapidly than the one of Gaspé and North Shore groups. Note that the North Shore sampling in the present study was extended to both eastern and western parts compared to a previous analysis which focused more on the western part [13]. This gives us a higher resolution and reveals differences that were not seen before since both parts have had a different colonization process. The observed IBD pattern is explained by the recent MRCA counts from five to ten meioses (Fig. 3B and Supplementary Table S2) which are more numerous in the Gaspé and North Shore groups than in the Sagueneans. In other words, the Sagueneans have less recent, but more distant common ancestors than other eastern groups. This is consistent with the close inbreeding being less important in Saguenay than among the Gaspé and the North Shore contemporary subjects (Supplementary Fig. S6) [27]. As MRCA can appear many times in this analysis and even though the Saguenean subjects descend from fewer founders than the other groups except GAC, they have more numerous MRCAs (after 13 meioses), which means that some of them appear very often, consistent with an expanding population due to a high birth rate and also observed in previous studies [29]. Another interesting ethnocultural group is the Gaspé Loyalists (GLO) which is genetically different from other Quebec groups on the second and the third PC (Supplementary Fig. S2). The GLO is among the groups with the lowest mean number of short IBD segments shared per pair, but they reach the second highest mean number of pairs sharing IBD segments (after the GAC group) above 27 cM. Indeed, GLO ancestors did not undergo a marked kinship increase around 1750 like Saguenay and GAC ancestors, they show an inbreeding increase after 1850 (Fig. 2B) which is consistent with the highest inbreeding values found at the 6–7 generations for GLO subjects (Supplementary Fig. S6) and corresponds to the appearance of the Gaspé groups differentiation (Supplementary Fig. S4). The GLO group comes from more numerous and diversified founders compared with GAC which could have affected the sharing of short IBD segments [35]. After their settlement, the GLO ancestors have remained quite isolated for more than 150 years as shown by their rapid inbreeding increase after 1850 (Fig. 2B), which could have exacerbated their sharing of long IBD segments. This is again consistent with previous findings on paternal and maternal lineages [31] and explains the particular IBD sharing among GLO subjects.

In this study, we show a similar pattern for the shared IBD segment lengths’ distribution and the cumulative number of genealogical MRCAs per subject pair (Fig. 3AB). Shared IBD segment length distribution depends on the number of common ancestors and the distance connecting both subjects to their common ancestor as it has been shown before using simulations on two individuals’ pairs [22]. The chance of transmitting a segment also depends on the GC of the common ancestor to both descendants [22]. Thus, common ancestors who contributed a lot to the present-day gene pool would be more susceptible to transmitting an IBD segment than those who contributed less. Usually, the closer the ancestors are to their descendants, the bigger their GC is (Fig. 3C). But in Saguenay, there are unusually great contributors among distant ancestors [29]. The number of MRCAs above 20 meioses is in the same order of magnitude for Saguenay as for Montreal and Quebec City subjects. However, the Saguenay ancestors’ GC above 10 meioses was higher and the resulting IBD sharing of shorter segments (less than 22 cM) is also higher. Sagueneans share more segments of less than 22 cM than any other group except GAC. In turn, GAC close common ancestors have a larger GC and they have the highest IBD sharing for all length bins even if their close MRCA counts (until 8 meioses) are similar to the other Gaspé groups and the North Shore subjects, suggesting that close MRCAs might have transmitted not only long, but also short IBD segments to their descendants.

Some limitations are present in this work. The genealogical completeness is not consistent across all groups (Supplementary Fig. S3) but was left uncorrected to retain two groups of Gaspé that would have been filtered out otherwise (Gaspé Loyalists and Channel Islanders) [13, 21]. This explains the aberrant curves in Fig. 3B, especially for Loyalists’ MRCA counts above 12 meioses. Also, note that if a structure was already present in the Quebec founders (for whom we reach the limit of the genealogy and we don’t know the parents), we do not have this information and we are unable to interpret its impact on the present-day population structure. We also reported an unequal number of participants across groups (Table 1). To overcome this, a bootstrap method was performed for specific genealogical analyses (Fig. 3BC). Finally, a generation gap was present between the Saguenay and the other groups (Table 1) that was not accounted for in Fig. 3 since the IBD sharing to be compared with genealogical MRCAs also includes this generation gap. For the other genealogical analyses presented in this study, this was not relevant since ancestors of each period were grouped regardless of the subjects’ generation.

In conclusion, genealogies are an invaluable tool to study the evolution of the population structure over time and to understand how the present-day genetic structure was shaped. We have shown that the Quebec population structure subdividing the Saguenay and Gaspé (especially Acadians and Loyalists) groups appeared early in the history of the province, even before the colonization of the Saguenay region. At that time, the Saguenay and GAC groups both experienced a marked average kinship and inbreeding increase until more recent times, when Saguenay reached a plateau and was almost joined by the other Gaspé groups. The resulting strong founder effect that occurred led to differences in the present-day IBD sharing and is linked to less numerous recent, but more numerous distant MRCAs for the Sagueneans compared to the GAC. Another understudied group, the GLO, was shown to have numerous recent MRCAs resulting in higher sharing of long IBD segments compared to all other groups except GAC. However, as their founders were more numerous and diversified and also due to the lower genetic contribution of their close MRCA, they did not go through a kinship increase as the Saguenay and GAC groups around 1750, but they did later and their resulting founder effect is less striking.

### Supplementary information

Supplemental material text summary

Supplemental material

Supplementary Table S1

## Data Availability

The 665 subjects’ genealogies and genotypes are available upon request to BALSAC at https://balsac.uqac.ca/ [12].

## Code availability

The code used for this study can be found in the following GitHub repository: https://github.com/laugag17/quebec_founder_pop.
