# Supplementary material for: Deciphering the genetic structure of the Quebec founder population using genealogies
Source: Eur J Hum Genet. 2023 Apr 4;32(1):91–7. doi: 10.1038/s41431-023-01356-2 (PMC10772069; doi:10.1038/s41431-023-01356-2)
Supplement: Supplementary file 1 — Supplemental material text summary [file 41431_2023_1356_MOESM1_ESM.pdf]

Supplementary Information text summary

In the ‘Supplemental material’ PDF file, you have access to Supplementary Figures S1 to S6 and the Supplementary Table S2.
The Supplementary Table S1 can be found in another excel file named ‘Supplementary_Table_S1’.
