# Supplementary material for: Deciphering the genetic structure of the Quebec founder population using genealogies
Source: Eur J Hum Genet. 2023 Apr 4;32(1):91–7. doi: 10.1038/s41431-023-01356-2 (PMC10772069; doi:10.1038/s41431-023-01356-2)
Supplement: Supplementary file 2 — Supplemental material [file 41431_2023_1356_MOESM2_ESM.pdf]

Supplemental Information

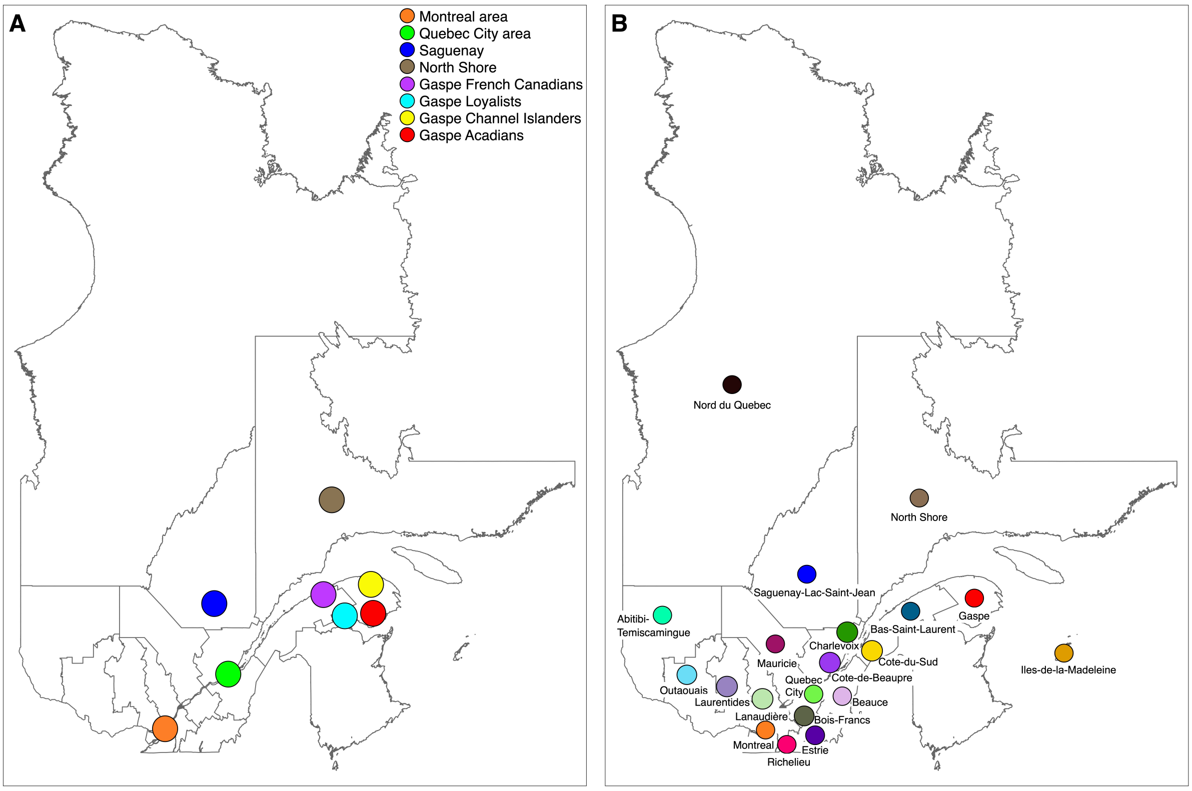

Supplementary Fig. S1. Geographical location of groups investigated in this study (A) and parents' regions of marriage used in figure 1 (B)

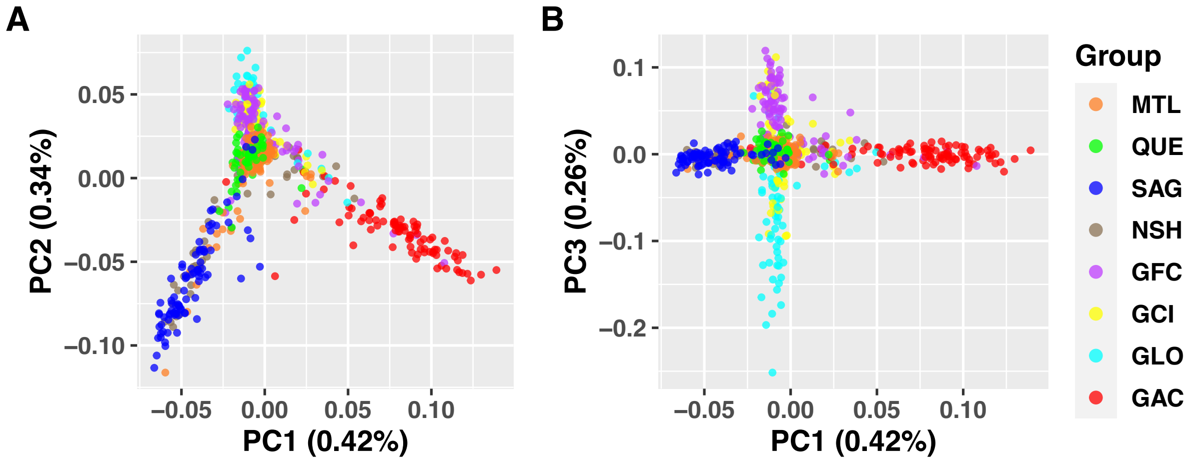

Supplementary Fig. S2. Principal component analysis of genotype data

Dots are colored according to the ethnocultural or regional group. Sample sizes are reported in Table 1 of the main text.

GAC=Gaspé Acadians ; GCI=Gaspé Channel Islanders ; GFC=Gaspé French Canadians ; GLO=Gaspé Loyalists ; MTL=Montreal ; NSH=North Shore ; QUE=Quebec City ; SAG=Saguenay.

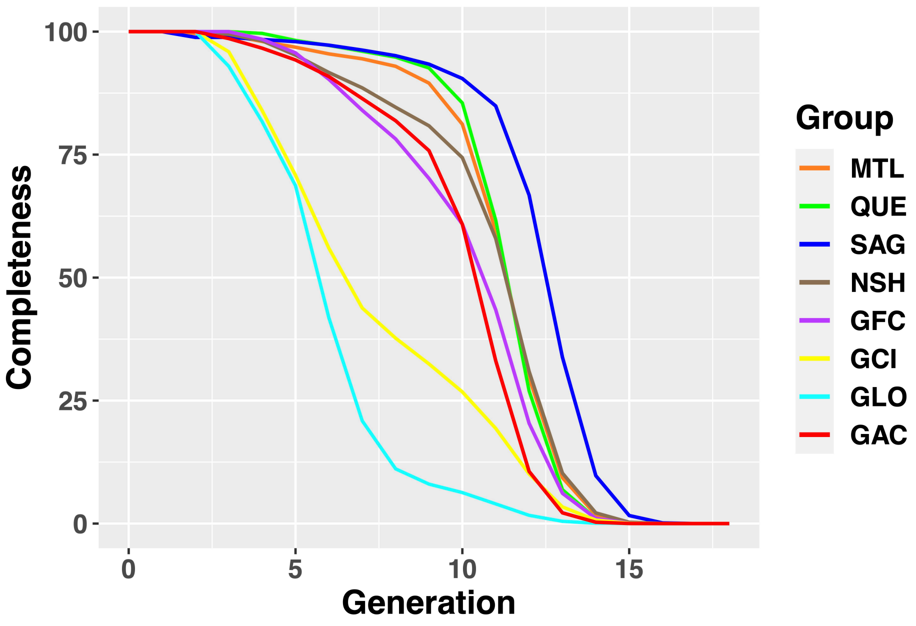

Supplementary Fig. S3. Contemporary groups' mean completeness per generation

The completeness is the proportion of ancestors present in the genealogy at each generation compared to the maximum possible number of ancestors. Sample sizes are reported in Table 1 of the main text.

GAC=Gaspé Acadians ; GCI=Gaspé Channel Islanders ; GFC=Gaspé French Canadians ; GLO=Gaspé Loyalists ; MTL=Montreal ; NSH=North Shore ; QUE=Quebec City ; SAG=Saguenay.

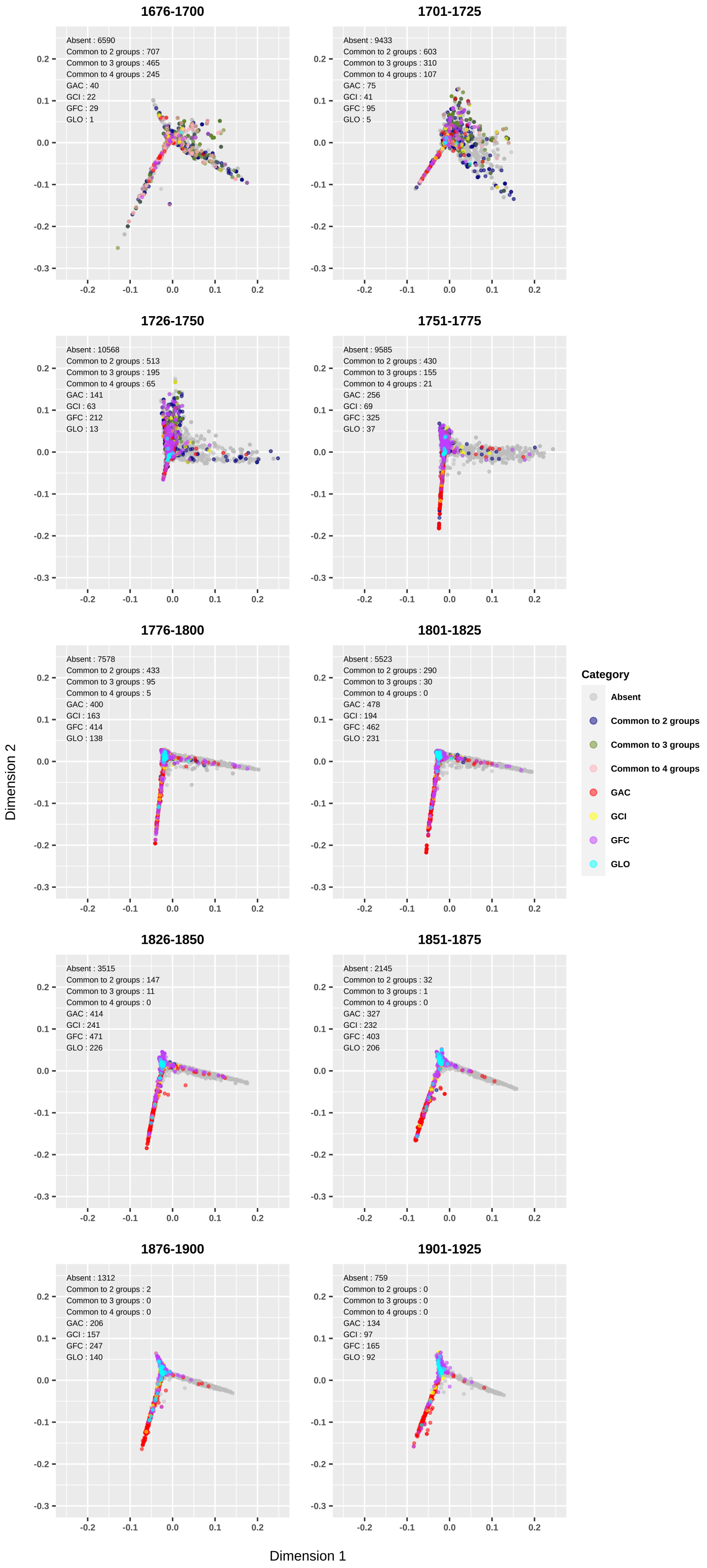

Supplementary Fig. S4. Multidimensional scaling (MDS) of the pairwise kinship coefficients of ancestors of Gaspé groups per 25-year period

MDS was performed on the pairwise kinship distance matrix, (i.e., 1-kinship coefficient) of ancestors whose parents were married at each period. The pairwise kinship coefficient was computed using the R GENLIB library at the maximal depth. Dots were colored according to the contemporary Gaspé group. If the ancestors happened to be the ancestor of more than one group, they were colored accordingly.

GAC=Gaspé Acadians ; GCI=Gaspé Channel Islanders ; GFC=Gaspé French Canadians ; GLO=Gaspé Loyalists.

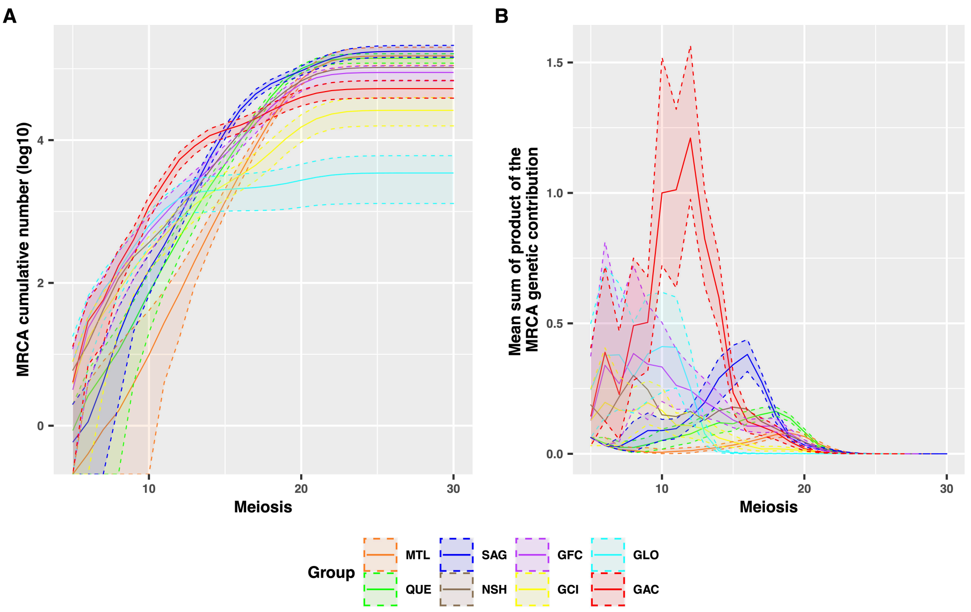

Supplementary Fig. S5. MRCA cumulative count (A) and genetic contribution (B) per meiosis within groups

The solid line represents the mean and the dashed lines are the maximum and minimum values of 1,000 bootstraps of 47 individuals.

GAC=Gaspé Acadians ; GCI=Gaspé Channel Islanders ; GFC=Gaspé French Canadians ; GLO=Gaspé Loyalists ; MTL=Montreal ; NSH=North Shore ; QUE=Quebec City ; SAG=Saguenay.

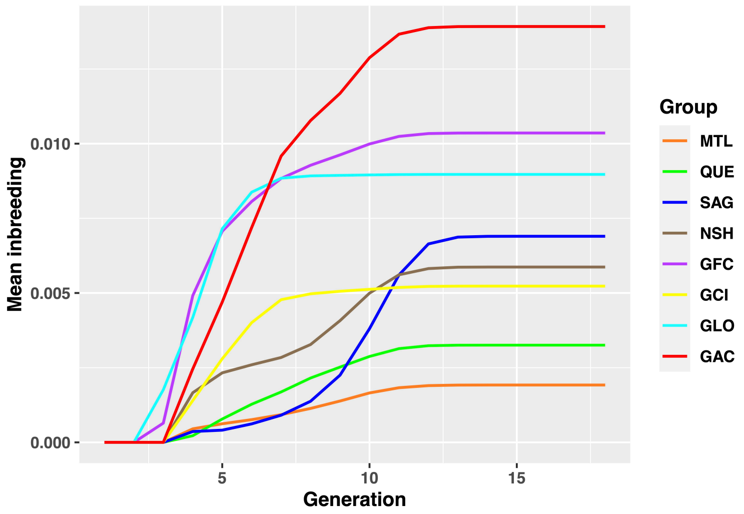

Supplementary Fig. S6. Mean inbreeding coefficient of contemporary subjects per group per generation

Mean inbreeding was calculated on the contemporary subjects of each group at each generation depth (x axis) using GENLIB. The inbreeding coefficient depends on the number of common ancestors present in both parents' genealogy. Close inbreeding (until ~4 generations) provides information on the choice of a spouse while distant inbreeding rather reflects the demographic history of the population. Sample sizes are reported in Table 1 of the main text.

GAC=Gaspé Acadians ; GCI=Gaspé Channel Islanders ; GFC=Gaspé French Canadians ; GLO=Gaspé Loyalists ; MTL=Montreal ; NSH=North Shore ; QUE=Quebec City ; SAG=Saguenay.

Supplementary Table S2. Cumulative MRCA counts
Mean of 1,000 bootstraps of 47 individuals.
GAC=Gaspé Acadians ; GCI=Gaspé Channel Islanders ; GFC=Gaspé French Canadians ; GLO=Gaspé Loyalists ; MTL=Montreal ; NSH=North Shore ; QUE=Quebec City ; SAG=Saguenay.

	**6**	**8**	**10**	**12**	**14**	**16**	**18**	**20**	**22**	**24**	**26**	**28**	**30**
MTL	0	2	10	72	534	3441	19239	70845	129160	148650	151048	151164	151165
QUE	3	11	73	428	2238	9779	40217	100058	134125	139772	140152	140154	140154
SAG	1	18	151	845	5701	26733	61315	93452	139673	168269	175136	175891	175902
NSH	14	115	367	1142	3756	12913	33438	67604	95445	103129	104070	104097	104097
GFC	23	136	529	1559	4048	10120	28829	61358	82731	87996	88586	88612	88612
GCI	17	77	292	783	1767	3214	6734	15264	23194	25801	26118	26119	26119
GLO	32	150	630	1535	1954	2100	2281	2740	3239	3430	3462	3463	3463
GAC	29	175	1181	5322	11577	16182	25855	39748	49457	52188	52614	52621	52621
